# Clinical significance of massive proteinuria in primary IgA nephropathy with and without nephrotic syndrome: a single center cohort study

**DOI:** 10.1080/0886022X.2023.2267138

**Published:** 2023-10-18

**Authors:** Ya Hu, Ziyuan Huang, Qianqian Cao, Bo Chen, Shungang Xu, Wenxian Qiu, Xiaohan You, Ji Zhang, Chaosheng Chen

**Affiliations:** a Department of Nephrology, The First Affifiliated Hospital of Wenzhou Medical University, Wenzhou, Zhejiang, PR China; b Institute of Chronic Kidney Disease, Wenzhou Medical University, Wenzhou, Zhejiang, PR China

**Keywords:** IgA nephropathy, nephrotic syndrome, proteinuria

## Abstract

**Background:**

Both primary IgA nephropathy (IgAN) with and without nephrotic syndrome (NS) can present massive proteinuria (24-h urinary protein ≥3.5 g/d). The clinical significance of massive proteinuria may be different in the two entities and needs further research.

**Methods:**

Data of 1870 patients with biopsy-proven IgAN in our hospital from January 2011 to December 2022 was retrospectively reviewed. A total of 242 IgAN patients with massive proteinuria were enrolled. Patients who presented with nephrotic syndrome at renal biopsy were included in the IgAN with NS cohort (IgAN-NS). The IgAN with nephrotic-range proteinuria cohort (IgAN-NR) consisted of 1:1 matched cases from the remaining according to age, gender, estimated glomerular filtration rate (eGFR) at baseline, and follow-up time. The clinical and pathological characteristics between the two cohorts were analyzed.

**Results:**

The IgAN-NS had a significantly higher proteinuria level than the IgAN-NR (*p* < .001). Cluster analysis revealed that proteinuria was associated with lipids in IgAN-NS, while it was associated with inflammatory indicators in IgAN-NR. When the complete remission of proteinuria (CR) was not achieved, the Kaplan–Meier analysis showed the prognosis of IgAN-NS was significantly worse than that of IgAN-NR (*p* = .04). Then, our GLMM model and line chart showed that the serum albumin level of the IgAN-NR was always evidently higher than that of the IgAN-NS while the significant difference in urinary albumin/creatinine ratio between the two cohorts gradually disappeared during the short-term follow-up (1 year). Moreover, the Cox regression analysis showed that the increased serum albumin was an independent protective factor for the poor outcomes (eGFR decreased from the baseline ≥ 30% continuously or reached end-stage renal disease [ESRD]).

**Conclusion:**

The IgAN-NS had poorer clinicopathologic manifestation than IgAN-NR, including severer massive proteinuria. When the CR was not achieved, the prognosis of IgAN-NS was inferior to that of the IgAN-NR.

## Introduction

The immunofluorescence (IF) manifestation of IgA nephropathy (IgAN) is well-established and is featured by the presence of IgA-dominant immune complexes in the renal mesangium [1]. However, under light microscope, IgAN can present with different histological patterns, including mesangial proliferative glomerulonephritis, focal segmental glomerulosclerosis, and crescentic glomerulonephritis [2]. As a result, IgAN can have diverse clinical manifestations such as hematuria, proteinuria, acute renal failure, and others [3]. Proteinuria is the second most common clinical features of IgAN after hematuria [4]. Nephrotic syndrome (NS), characterized by severe proteinuria, low serum albumin levels, and edema, occurs in approximately 5–10% of IgAN patients. In some cases, IgAN with NS behaves similarly to minimal change disease (MCD) [5,6]. However, in some IgAN patients with NS, the massive proteinuria does not relieve with corticosteroid treatment, and the renal function gradually deteriorates, indicating a different response to corticosteroids compared to MCD [7]. Therefore, it is important to study the clinicopathological and prognostic features of IgAN patients with NS. Previous research has reported that some IgAN patients may present with massive proteinuria but without low levels of proteins in the blood (hypoalbuminemia) [7]. Li et al. [5] compared these patients to IgAN patients with NS, and demonstrated no obvious difference in prognosis between the two groups. They speculated that hypoalbuminemia was not a significant factor influencing the outcomes in IgAN patients with massive proteinuria. However, these studies did not clearly document the changes in proteinuria over time during follow-up, and the clinical significance of massive proteinuria in the two patient groups may be different, necessitating further investigation. Previous research [8] has also shown that early resmission of proteinuria, especially spontaneous remission within a short period, can significantly improve the prognosis of IgAN patients with NS. Therefore, it is crucial to consider the clinical significance of proteinuria and its impact on prognosis. Herein, two cohorts, namely, IgAN patients with NS (IgAN-NS) and IgAN patients with nephrotic-range proteinuria (IgAN-NR), were selected according to the results of 24-h urinary protein (UP) quantification and serum albumin levels during renal biopsy. Propensity score matching [9] was performed to ensure comparability between the two cohorts in terms of gender, age, estimated glomerular filtration rate (eGFR), and follow-up duration. The study aimed to compare the prognostic and clinicopathological differences between the IgAN-NS and IgAN-NR cohorts, as well as analyze the clinical significance of proteinuria and its influence on prognosis. Also, the larger sample size of this research facilitate systematic reviews. The findings from this study can provide valuable insights for the clinical diagnosis and treatment of IgAN.

## Methods

### Patient profiles

A retrospective review of data from 1870 cases of biopsy-proven IgAN at our hospital from January 2011 to December 2022 was conducted. Among them, 242 patients met the selection criteria and were recruited into this study. The selection criteria were as follows: (1) absence of other primary/secondary glomerular diseases (e.g., chronic advanced liver disease, systemic lupus erythematosus, Henoch-Schonlein purpura, etc.); (2) presence of massive proteinuria (24-h UP ≥3.5 g/d) during renal biopsy; (3) a follow-up duration of >3 months after the renal biopsy; and (4) availability of complete clinicopathological data required for this study. Of the 242 enrolled patients, 111 patients who presented with NS (defined as both 24-h UP ≥3.5 g/d and serum albumin <30 g/L) [10] at the renal biopsy were included in the IgAN-NS group. The remaining 131 patients, who exhibited massive proteinuria without hypoalbuminemia, were included in the IgAN-NR group. For the IgAN-NS group, a cohort was established by matching 1:1 cases from the IgAN-NR group based on gender, age, follow-up period, and renal function. The prognostic and clinicopathological differences between the two cohorts were determined in this single-center retrospective cohort study, which was approved by the Ethics Committee of the the First Affiliated Hospital of Wenzhou Medical University. All data were collected from the information system of the hospital, and patient-identifiable information was anonymized.

### Data collection

During the initial renal biopsy, data regarding age, gender, weight (kg), height (m), hemoglobin (Hb), blood pressure (mmHg), serum creatinine (s-Cr), uric acid, serum albumin, triglyceride, total cholesterol, LDL-cholesterol, HDL-cholesterol, C-reactive protein (CRP), fibrinogen, serum IgA, 24-h UP, and urinary albumin/creatinine ratio were collected and considered as baseline measurements. During the follow-up period, additional data on medication usage (e.g., immunosuppressants, steroids, and renin-angiotensin-aldosterone system blockade), urinary albumin/creatinine ratio, serum creatinine, and serum albumin levels were collected.

All renal biopsy specimens were examined using IF and light microscopy (LM). Approximately 33% of the samples were examined using electron microscopy (EM). The pathologic diagnosis based on LM findings was determined by 2 senior pathologists following the Oxford classification criteria [11]. This classification includes endocapillary hypercellularity (E0/1), mesangial hypercellularity (M0/1), tubular atrophy and interstitial fibrosis (T0/1/2), segmental glomerulosclerosis (S0/1), and crescent formation (C0/1). Additionally, statistical analysis was performed on the percentage of glomerulosclerosis. For the immunofluorescence analysis, frozen sections were stained using the direct immunofluorescent approach. The staining intensities of fibrinogen, complement, and immunoglobulin deposits were assessed based on the IF results. The staining intensities were categorized as follows: −, +, ++, +++ and ++++ [12].

### Definitions

Throughout the follow-up period following the initial renal biopsy, the average urinary albumin/creatinine ratio was calculated for each 6-month interval, considering the part of less than 6 months as one interval. This calculated value was referred to as the time-average albumin/creatinine ratio (TA-UACR). Complete remission of proteinuria (CR) was defined as having a TA-UACR consistently below 300 mg/g at least once during the follow-up. Partial remission of proteinuria was referred to a ≥ 50% decrease in TA-UACR from the baseline, with a value remaining below 3500 mg/g at least once during the follow-up period [7]. The eGFR was detected using the CKD-EPI Creatinine Equation (2021) [13]. The composite endpoint was described as either a continuous decline in eGFR from a baseline of at least 30% [14] or reaching ESRD.

### Statistical analysis

For numerical variables, the mean (standard deviation – SD) or median [interquartile range – IQR] was used for presentation, and their comparisons were performed using variance analysis or the Kruskal–Wallis rank test, depending on the distribution of the data. Categorical data were expressed as counts (%) and analyzed using Pearson’s chi-squared test. To account for multiple comparisons, the Benjamini-Hochberg method was applied for correction. The correlation coefficient (Pearson’s method for linear data) was calculated to generate a heatmap and visualize the relationships. Hierarchical clustering was then employed to create a network that displays the clustering relationships [15]. The prognosis was assessed using the Kaplan–Meier method (KM), and the log-rank test was used to analyze the differences between groups. To examine the relationship between repeatedly measured urinary albumin/creatinine ratio and serum albumin in the two cohorts, generalized linear mixed-effects models (GLMM) were constructed [16]. The GLMM accounts for random effects (e.g., individual differences), and Analysis of Variance (ANOVA) was employed for comparisons. Adjusted marginal means and standard errors (SEs) of urinary albumin/creatinine ratio and serum albumin were plotted using line charts to illustrate the trend in a direct manner. Multivariate-Cox-proportional-hazards models were used to identify independent factors affecting the prognostic outcomes. The models were established based on factors showing statistical significance in univariate-Cox-regression analysis, using forward-backward (FB) stepwise methods and optimizing with the Akaike-information-criterion. All reported *p*-values were two-tailed, and a significance level of *p* < .05 was deemed statistically significant. Missing values were handled using multiple imputations [17] (details on the percentage of missing data was shown in Table S1). The data analysis and visualization were conducted using R v4.1.3 [18,19] and R packages (such as *pheatmap, nlme, and surveminer*).

## Results

### Comparison of clinicopathological manifestations between IgAN-NS and IgAN-NR

From January 2011 to December 2022, there were 1870 biopsy-proven IgAN cases at our hospital (Supplemental Figure 1). Among them, 283 IgAN patients who presented with massive proteinuria were selected (126 IgAN-NS and 157 IgAN-NR). The IgAN-NS cohort had a median age of 41.0 years at the time of diagnosis, and there was a male-to-female ratio of 1.3–1. Moreover, 41 cases were excluded due to short follow-up duration or incomplete data, and the remaining 242 cases (111 IgAN-NS and 131 IgAN-NR) were ultimately included in this study. Subsequently, the IgAN-NR cohort was matched with the IgAN-NS cohort in a 1:1 ratio using propensity score matching. The findings presented in Table 1 highlight the disparities between the IgAN-NS (right-column) and IgAN-NR (left-column) cohorts. Following the propensity score matching process, which accounted for gender, age, follow-up time, and eGFR, no obvious differences were observed in the demographic variables between the two cohorts. The IgAN-NS had significantly decreased levels of average hemoglobin (*p* < .001), serum albumin (*p* < .001), and triglyceride (*p* = .005). The average total cholesterol (*p* < .001), HDL-cholesterol (*p* = .01), LDL-cholesterol (*p* < .001), fibrinogen (*p* = .008), CRP (*p* = 0.006), 24-h UP (*p* < .001), urinary red blood cell (RBC) count (*p* = .04) and white blood cell (WBC) count (*p* = .002) level were remarkably higher in IgAN-NS. A higher proportion of patients with IgAN-NS received corticosteroid treatment (*p* < .001). Composite endpoints were observed in 72 cases, including 38 IgAN-NS cases and 34 IgAN-NR cases, and the incidence was not remarkably different between the two cohorts. A total of 181 cases achieved remission of proteinuria, including 93 cases in the IgAN-NS group (54 CR and 39 PR) and 88 cases in the IgAN-NR group (49 CR and 39 PR), and the incidence of remission was not markedly different between the two cohorts. The pathologic results showed an average of 20.2 glomeruli per specimen, with an average proportion of glomerulosclerosis of 35.5%. The IgAN-NS exhibited markedly increased and decreased proportions of M1 (59% vs 40.3%, *p* = .04) and glomerulosclerosis (32% vs 39%, *p* = .04), respectively. The IF results showed that IgA was the predominant or co-dominant immunoglobulins accumulated on the capillary walls and/or glomerular mesangial areas, with varying intensities ranging from + to ++++. However, there was no significant difference in the intensity of these depositions between the two cohorts. The EM results showed varying degrees of segmental foot process effacement in some cases, and no obvious hump-like subendothelial deposits were seen.

**Table 1.** Comparison of the clinicopathological manifestations between IgAN-NS and IgAN-NR.

	IgAN-NR	IgAN-NS	*p*
*N*	111	111	
Age, y	41.00 [33.50, 50.00]	41.00 [27.00, 54.00]	.7
Male	63 (56.8)	55 (49.5)	.3
Follow-up duration,m	39.53 [17.95, 67.58]	36.50 [13.98, 72.27]	.5
eGFR	61.49 (29.46)	61.68 (37.37)	.9
Body mass index	30.62 (40.21)	23.74 (36.65)	.1
Systolic blood pressure, mmHg	128.90 (13.68)	129.11 (14.28)	.9
Diastolic blood pressure, mmHg	80.15 (8.21)	78.98 (11.48)	.4
Mean arterial pressure, mmHg	123.11 (11.79)	122.02 (14.58)	.6
Hemoglobin, g/L	126.67 (21.06)	113.49 (23.16)	<.001
Serum creatinine, mg/dL	1.96 (2.20)	2.21 (2.60)	.4
Serum albumin, g/L	35.74 (4.58)	24.95 (3.96)	<.001
Total cholesterol, mmol/L	5.75 (1.60)	6.91 (2.42)	<.001
Triglyceride, mmol/L	2.93 (2.15)	2.26 (1.26)	.005
HDL-cholesterol, mmol/L	1.06 (0.27)	1.21 (0.53)	.01
LDL-cholesterol, mmol/L	3.14 (1.06)	4.13 (1.57)	<.001
Uric acid, mmol/L	420.76 (97.55)	409.30 (127.72)	.5
Fibrinogen, g/L	4.34 (1.05)	4.79 (1.40)	.008
C-reactive protein, g/L	4.10 (5.33)	10.77 (24.89)	.006
Serum IgA, g/L	3.48 (1.22)	3.22 (1.33)	.1
Proteinuria, g/24 h	5.66 (2.29)	7.35 (3.17)	<.001
Urine red blood cell count, RBC/μL	157.49 [43.26, 499.54]	297.90 [94.48, 836.96]	.04
Urine white blood cell count, WBC/μL	125.50 [65.41, 261.33]	202.56 [107.36, 467.60]	.002
Composite endpoint	34 (30.6)	38 (34.2)	.7
CR	49 (44.1)	54 (48.6)	.7
PR	39 (35.1)	39 (35.1)	1
Therapy			
Renin-angiotensinsystem inhibito	100 (90.1)	94 (84.7)	.3
Steroid	74 (66.7)	96 (86.5)	.001
Immunosuppressant	57 (51.4)	48 (43.2)	.3
Renal biopsy			
Number of glomeruli	19.58 (10.61)	20.81 (10.28)	.4
Proportion of glomerulosclerosis, %	39.91 (27.32	32.52 (23.43)	.04
Oxford classification			
M1	27 (40.3)	46 (59.0)	.04
E1	37 (51.4)	48 (59.3)	.4
S1	56 (80.0)	62 (77.5)	.9
T			.3
T1	25 (34.7)	20 (24.7)	
T2	25 (34.7)	28 (34.6)	
C1	68 (61.3)	68 (61.3)	1
Immunofluorescence			
IgA			.7
+	40 (36.0)	36 (32.4)	
++	8 (7.2)	13 (11.7)	
+++	62 (55.9)	61 (55.0)	
++++	1 (0.9)	1 (0.9)	
IgM			.9
+	34 (30.6)	30 (27.0)	
++	12 (10.8)	14 (12.6)	
+++	1 (0.9)	2 (1.8)	
IgG			.7
+	7 (6.3)	7 (6.3)	
++	5 (4.5)	2 (1.8)	
+++	1 (0.9)	1 (0.9)	
C3			.3
+	42 (37.8)	36 (32.4)	
++	36 (32.4)	28 (25.2)	
+++	24 (21.6)	34 (30.6)	
C4			1
++++	1 (0.9)	0 (0.0)	

Numerical variables are reported as means (SD) or medians [IQR], while categorical variables are presented as counts (%). The composite endpoint was referred to a continuous decline in estimated eGFR from baseline of ≥30% or reaching ESRD.

IgAN-NS refers to IgAN with NS, while IgAN-NR represents IgAN with nephrotic-range proteinuria. E1 signifies endocapillary hypercellularity, M1 denotes mesangial hypercellularity, T1-2 refers to the severity of interstitial fibrosis/tubular atrophy, and S1 indicates segmental glomerulosclerosis/adhesion. C1 indicates the presence of crescent. CR signifies complete remission of proteinuria, while PR denotes partial remission of proteinuria.

^a^ P-value was adjusted for multiple comparisons.

**Figure 1.**
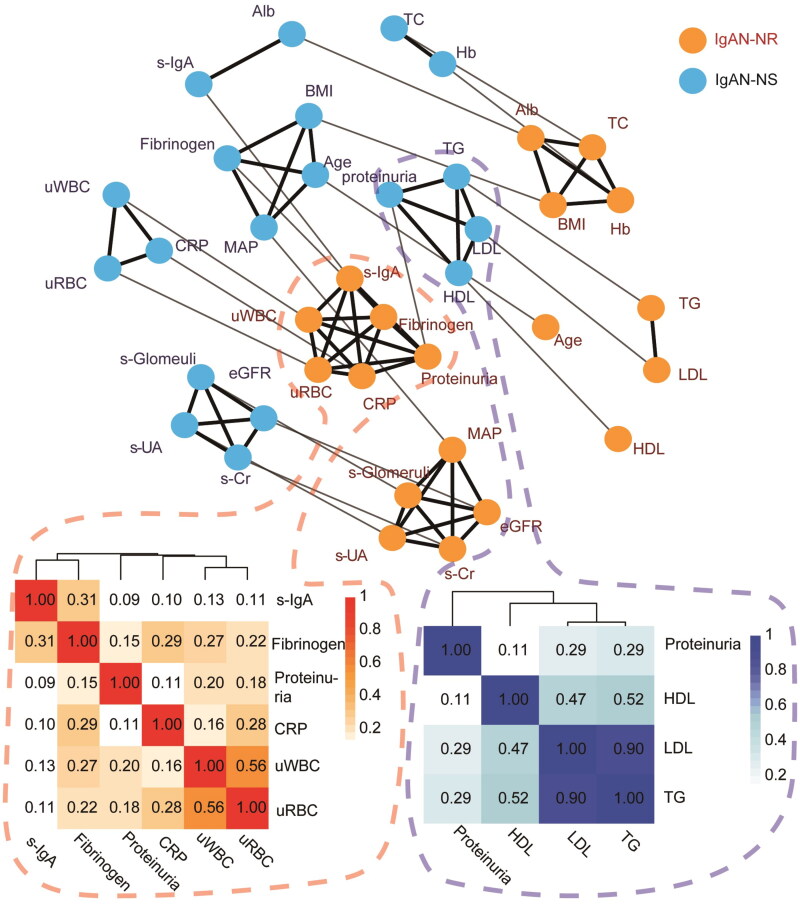
The network diagram Illustrates the clustering relationship of clinical factors within IgAN-NS and IgAN-NR. Each factor is represented by a node, with orange nodes representing IgAN-NS and light blue nodes representing IgAN-NR. Nodes that are linked by solid black lines indicate a stronger correlation and form a cluster, while nodes that are linked by solid gray lines represent the same factors from various groups (assigned a small weight for the same factors and a larger weight for different factors). to further analyze the clusters, including the factor ‘proteinuria’ in both cohorts, a cluster analysis was conducted using a heatmap. The heatmaps depict the correlation coefficients (pearson’s-correlation-coefficient for linear data) of the clinical factors within IgAN-NS and IgAN-NR. The color scale below the heatmap indicates that darker shades represent positive correlations, whereas lighter shades represent negative correlations. Factors with similar colors are considered to be related or share similar correlations. Alb: serum albumin; BMI: body mass index; eGFR: estimated glomerular filtration rate; Hb: hemoglobin; HDL: HDL-cholesterol; LDL: LDL-cholesterol; MAP: mean blood pressure; s-Cr: serum creatinine; TC: total cholesterol; TG: triglyceride; s-UA: serum uric acid; CRP: C-reactive protein; s-IgA: serum IgA; uRBC: urinary red blood cell counts; uWBC: urinary white blood cell counts; s-Glomeruli: proportion of glomerulosclerosis; IgAN-NS: the IgAN with NS; IgAN-NR: the IgAN with nephrotic-range proteinuria.

The network diagram and heatmap (Figure 1) representing the clinical parameters of the two cohorts demonstrated notable differences in the clustering pattern related to proteinuria levels between IgAN-NS and IgAN-NR. In the IgAN-NS group, proteinuria levels showed a positive association with lipid markers such as triglyceride, HDL-cholesterol, and LDL-cholesterol. Conversely, in the IgAN-NR group, proteinuria levels were correlated with inflammatory indicators, including serum IgA, fibrinogen, CRP, urine RBC count, and urine WBC count. These findings indicate distinct associations between proteinuria and lipid metabolism in IgAN-NS, while IgAN-NR appears to be more closely linked to inflammation-related markers.

### Differences in prognostic outcomes between IgAN-NS and IgAN-NR and risk factors

**Figure 2.**
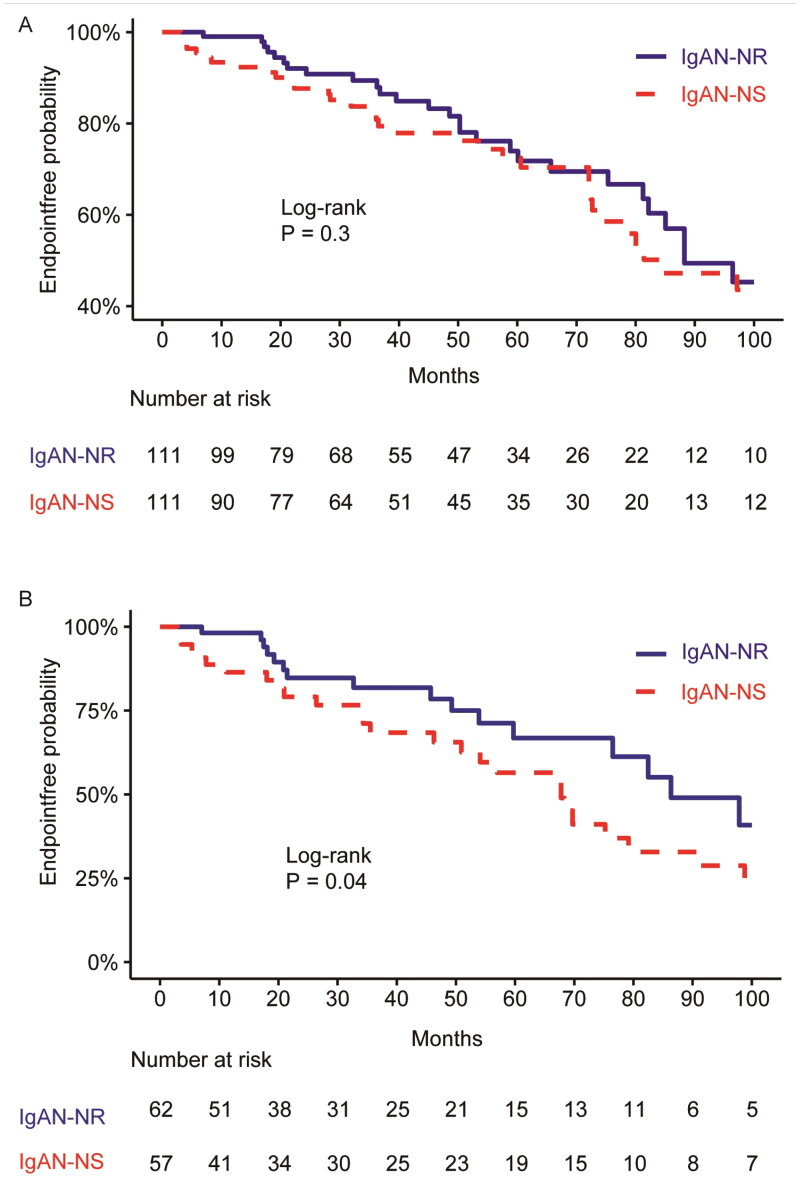
(A) Kaplan-Meier curve displayed the prognosis of the IgAN-NS and IgAN-NR cohorts (*p* = .3 [Log-rank test]). (B) Kaplan–Meier curve displayed the prognosis of the IgAN-NS and IgAN-NR without CR (*p* = .04 [log-rank]). The endpoint was defined as either a reduction of ≥30% in baseline eGFR or the development of ESRD. CR: complete remission of proteinuria; IgAN-NS: the IgAN with NS; IgAN-NR: the IgAN with nephrotic-range proteinuria.

The median follow-up time was 38.0 months, ranging from 6 to 96 months. After matching, there were no obvious differences in baseline demographic variables and renal function between the two cohorts. When considering a composite endpoint of a continuous decline in eGFR from a baseline of ≥30% or reaching ESRD, the KM curve (Figure 2(A)) displayed no obvious differences in the survival rate without composite endpoints between the two cohorts (IgAN-NS vs. IgAN-NR: HR = 1.27; 95% CI =0.79–2.01; log-rank, *p* = .3).

**Figure 3.**
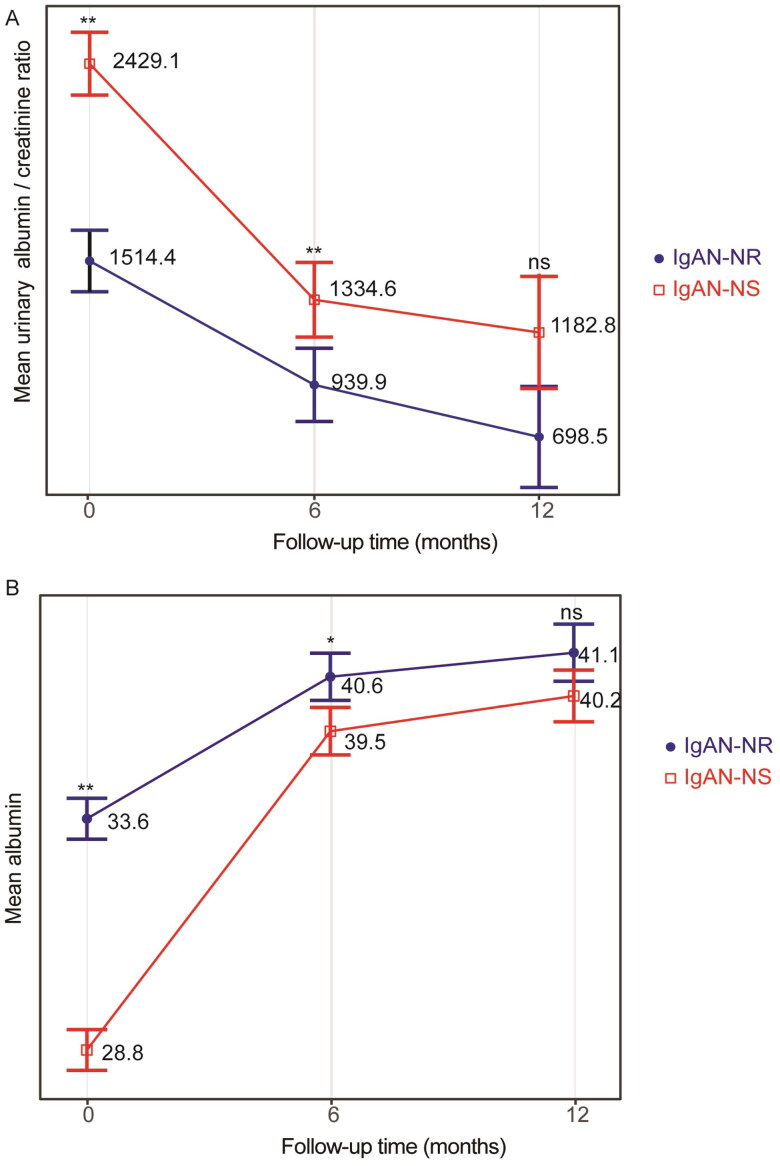
(A) Estimated means and corresponding SEs of urinary albumin/creatinine ratio were Adjusted for within the follow-up period using GLMM in IgAN-NS and IgAN-NR. (B) Estimated means and corresponding SEs of albumin levels were estimated and compared between IgAN-NS and IgAN-NR within the follow-up period using GLMM. The ANOVA results of the GLMM are presented in Table 5. Statistical significance levels are indicated in the plot as ‘*’, which represents *p* < .05, and ‘**’, representing *p* < .01. IgAN-NS: the IgAN with NS; IgAN-NR: the IgAN with nephrotic-range proteinuria; ns: no statistical difference.

The GLMM (Tables 5 and 6) indicated a clear decrease in urinary albumin/creatinine ratio and an increase in serum albumin in both cohorts during the follow-up period. Significant differences of urinary albumin/creatinine ratio were observed between the two cohorts. The between-group variation interacted with the duration of follow-up, as indicated by the line chart, which showed no significant between-group variation at the 1-year follow-up (Figure 3). In the multivariate Cox regression analysis (Table 2), increased serum uric acid (*p* = .01), presence of S1 (segmental glomerulosclerosis) (*p* = .03), and presence of M1 (mesangial hypercellularity) (*p* = .04) were identified as independent risk factors. On the other hand, increased HDL-cholesterol (*p* = .03) was identified as an independent protective factor.

**Table 2.** Factors associated with reaching the endpoint in the IgN-NS and IgAN-NR.

Factors	HR	CI	*p*	HR	CI	*p*
Age	1.01	0.99–1.02	.4			
Serum albumin	0.99	0.96–1.03	.7			
Body mass index	1.01	0.98–1.04	.4			
C1	1.12	0.69–1.83	.6			
E1	0.69	0.37–1.28	.2			
Fibrinogen	1.13	0.94–1.37	.2			
Hemoglobin	0.77	0.43–1.37	.4			
HDL-cholesterol	0.4	0.17–0.95	.04	0.29	0.06–0.84	0.03
LDL-cholesterol	0.93	0.77–1.12	.4			
Mean arterial pressure	1.01	1–1.03	.1			
M1	2.06	1.02–4.13	.04	1.81	1.22–5.33	.04
Proteinuria	1.07	0.99–1.15	.09	1.07	0.99–1.16	.09
Serum creatinine	0.99	0.91–1.09	.9			
Male	1.5	0.92–2.43	.1			
S1	2.6	0.92–7.33	.07	2.97	0.90–7.41	.06
Total cholesterol	1.06	0.94–1.2	.3			
Triglyceride	0.97	0.86–1.1	.7			
T1	1.67	1.15–5.22	.2			
T2	0.74	0.39–1.4	.4			
Serum IgA	1.12	0.69–1.83	.6			
Uric acid	1.17	1.04-1.32	.01	1.14	1.01-1.29	.01
Urine red blood cell count	0.97	0.88–1.06	.5			
Renin-angiotensinsystem inhibito	0.99	0.97–1.01	.3			
Steroid	0.98	0.9–1.07	.7			
Immunosuppressant	0.7	0.37–1.33	.3			

The endpoint was defined as a continuous decline in eGFR from baseline of ≥30% or reaching ESRD. Factors that showed statistical significance (*p* < .1) in univariate-Cox-regression analysis were selected for inclusion in the multivariate-Cox-regression model. The FB stepwise method using AIC was employed to choose the best model with a minimum AIC value.

E1: signifies endocapillary hypercellularity; M1: denotes mesangial hypercellularity; T1-2: refers to the severity of interstitial fibrosis/tubular atrophy; S1: indicates segmental glomerulosclerosis/adhesion; C1: signifies the presence of a crescent; HR: represents the hazard ratio; CI: stands for confidence interval; AIC: represents the Akaike information criterion.

### Comparison of clinicopathological manifestations and prognosis between IgAN-NS and IgAN-NR without CR

**Figure 4.**
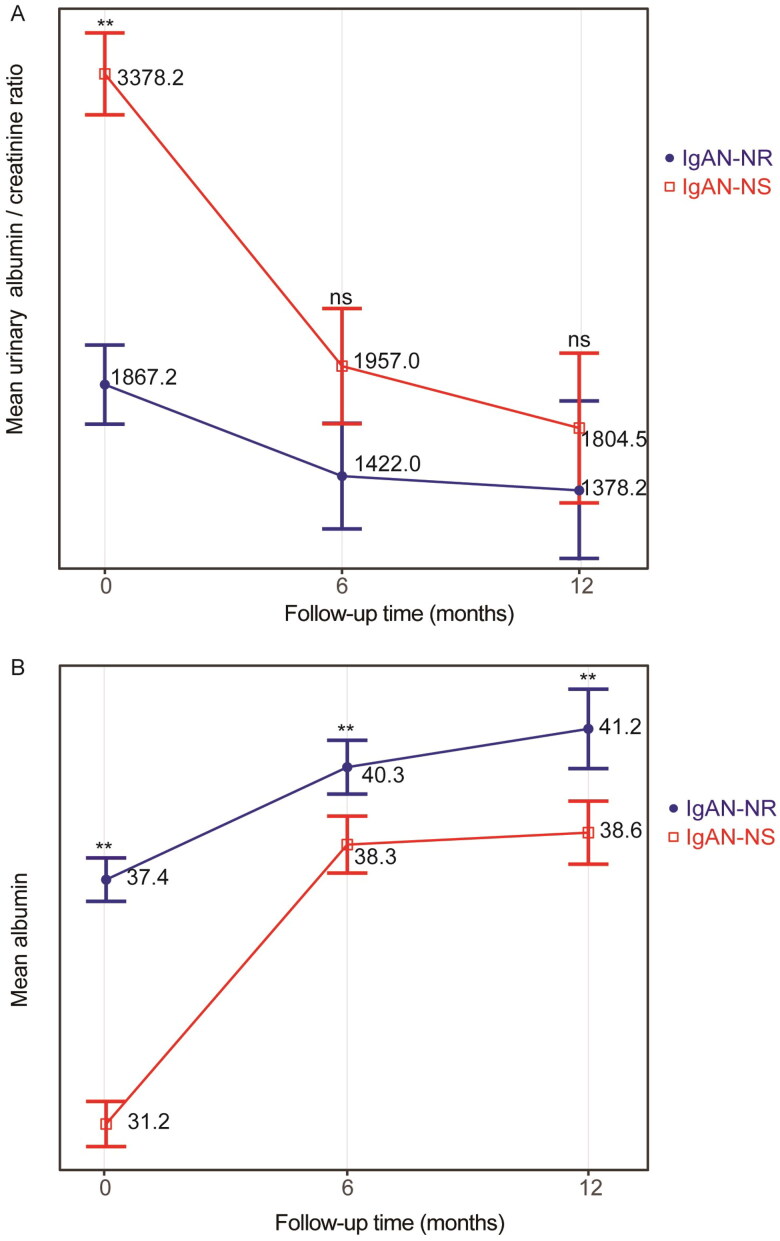
(A) Estimated means and corresponding SEs of urinary albumin/creatinine ratio between IgAN-NS and IgAN-NR without CR within the follow-up in GLMM. (B) Estimated means and corresponding SEs of albumin between IgAN-NS and IgAN-NR without CR within the follow-up in GLMM. The ANOVA results of the GLMM are presented in Table 5. Statistical significance levels are indicated in the plot as ‘*’, which represents *p* < .05, and ‘**’, representing *p* < .01. IgAN-NS: the IgAN with NS; IgAN-NR: the IgAN with nephrotic-range proteinuria; ns: no statistical difference; CR, complete remission of proteinuria.

We conducted a review of the follow-up data for 222 patients with IgAN-NS and IgAN-NR. Among them, 119 patients who did not achieve CR were included, comprising 57 cases in the IgAN-NS group and 62 cases in the IgAN-NR group. Although not matched, there were no significant differences in gender, age, follow-up period, and eGFR between the two cohorts (Table 3). Among the patients without CR, the IgAN-NS group exhibited significantly lower average levels of hemoglobin (*p* < .001), serum albumin (*p* < .001), and triglycerides (*p* = .03). Conversely, the average levels of total cholesterol (*p* = .002), HDL-cholesterol (*p* = .01), LDL-cholesterol (*p* < .001), fibrinogen (*p* = .005), serum IgA (*p* = .02), 24-h UP (*p* < .001), urinary RBC count (*p* = .04), and urinary WBC count (*p* = .008) were significantly higher in the IgAN-NS group. Furthermore, the IgAN-NS group exhibited a markedly higher proportion of M1 (mesangial hypercellularity) compared to the IgAN-NR group (72.2% vs. 38.9%, *p* = .009). Additionally, a larger proportion of patients in the IgAN-NS group received corticosteroid treatment. When considering a composite endpoint of a continuous decline in eGFR from baseline of ≥30% or reaching ESRD, the KM curve demonstrated a remarkably lower survival rate without composite endpoints in the IgAN-NS group (log-rank, *p* = .04; Figure 2(B)). In the multivariate Cox regression analysis (Table 4), the presence of M1 was identified as an independent risk factor, while increased serum albumin was found to be an independent protective factor. The GLMM (Table 7) demonstrated a significant decrease in urinary albumin/creatinine ratio for both cohorts during the follow-up, with significant differences between the two groups. The between-group variation interacted with the duration of follow-up, as indicated by the line chart, which showed no significant between-group variation at the 1-year follow-up (Figure 4). Nevertheless, the GLMM (Table 8) revealed a notable increase in serum albumin levels for both cohorts during the follow-up, with a notable difference observed between the two groups. The line chart indicated that the IgAN-NR group exhibited markedly higher serum albumin levels compared to the IgAN-NS group during the 1-year follow-up.

**Table 3.** Comparison of the clinicopathological manifestations between IgAN-NS and IgAN-NR without CR.

	IgAN-NR	IgAN-NS	*p*
*N*	62	57	
Age, y	42.50 [36.00, 50.50]	40.00 [27.00, 52.00]	.3
Male	35 (56.5)	26 (45.6)	.3
Follow-up duration, m	40.85 (33.78)	42.05 (35.47)	.9
eGFR	57.34 (26.50)	52.49 (33.34)	.4
Body mass index	31.52 (40.45)	24.38 (3.80)	.2
Systolic blood pressure, mmHg	130.78 (12.39)	131.27 (14.54)	.8
Diastolic blood pressure, mmHg	81.81 (8.18)	79.95 (8.34)	.2
Mean arterial pressure, mmHg	125.41 (11.28)	123.71 (11.94)	.4
Hemoglobin, g/L	125.91 (22.33)	110.05 (24.95)	<.001
Serum creatinine, mg/dL	1.90 (1.77)	2.49 (3.04)	.1
Serum albumin, g/L	35.98 (4.57)	25.32 (3.50)	<.001
Total cholesterol, mmol/L	5.63 (1.34)	6.69 (2.17)	.002
Triglyceride, mmol/L	3.01 (1.91)	2.33 (1.43)	.03
HDL-cholesterol, mmol/L	1.03 (0.28)	1.15 (0.51)	.1
LDL-cholesterol, mmol/L	3.05 (0.99)	3.92 (1.47)	<.001
Uric acid, mmol/L	423.03 (99.63)	417.89 (135.43)	.8
Fibrinogen, g/L	4.33 (1.14)	5.00 (1.45)	.005
C-reactive protein, g/L	4.03 (5.99)	10.94 (28.41)	.06
Serum IgA, g/L	3.56 (1.23)	3.05 (1.05)	.02
Proteinuria, g/24 h	5.62 (2.05)	7.50 (3.31)	<.001
Urine red blood cell count, RBC/μL	163.10 [27.45, 540.72]	302.12 [126.75, 515.85]	.04
Urine white blood cell count, RBC/μL	127.15 [64.91, 232.81]	205.97 [107.36, 545.53]	.008
Composite endpoint	21 (33.9)	28 (49.1)	.1
Therapy			
Renin-angiotensinsystem inhibitor	54 (87.1)	44 (77.2)	.2
Steroid	37 (59.7)	51 (89.5)	<.001
Immunosuppressant	27 (43.5)	29 (50.9)	.5
Oxford classification			
M1	14 (38.9)	26 (72.2)	.009
E1	18 (43.9)	20 (55.6)	.4
S1	33 (84.6)	33 (91.7)	.6
T			.5
T1	14 (34.1)	13 (36.1)	
T2	16 (39.0)	17 (47.2)	
C1	41 (66.1)	34 (59.6)	.6
Immunofluorescence			
IgA			.6
+	26 (41.9)	23 (40.4)	
++	3 (4.8)	5 (8.8)	
+++	33 (53.2)	28 (49.1)	
++++	0 (0.0)	1 (1.8)	
IgM			.7
+	20 (32.3)	20 (35.1)	
++	5 (8.1)	8 (14.0)	
+++	1 (1.6)	1 (1.8)	
IgG			.6
+	5 (8.1)	4 (7.0)	
++	1 (1.6)	0 (0.0)	
C3			.4
+	29 (46.8)	23 (40.4)	
++	19 (30.6)	13 (22.8)	
+++	9 (14.5)	15 (26.3)	

Numerical variables are reported as means (SD) or medians [IQR], while categorical variables are presented as counts (%). The composite endpoint was referred to a continuous decline in eGFR of ≥30% from the baseline or reaching ESRD.

IgAN-NS: IgAN with NS; IgAN-NR: IgAN with nephrotic-range proteinuria; E1: endocapillary hypercellularity; M1: mesangial hypercellularity; T1-2: severity of interstitial fibrosis/tubular atrophy; S1: segmental glomerulosclerosis/adhesion; C1: presence of crescent; CR: complete remission of proteinuria; PR:partial remission of proteinuria. a *p*-value was adjusted for multiple comparison.

**Table 4.** Factors associated with reaching the endpoint in the IgAN-NS and IgAN-NR without CR.

Factors	HR	CI	*p*	HR	CI	*p*
Age	1	0.98–1.02	1			
Serum albumin	0.22	0.11–0.47	<.001	0.21	0.1–0.45	<.001
Body mass index	1.01	0.98–1.05	.4			
C1	1.05	0.57–1.92	.9			
E1	1.42	0.62–3.24	.4			
Fibrinogen	1.96	0.82–4.7	.1			
Hemoglobin	0.97	0.92–1.02	.2			
HDL-cholesterol	0.59	0.22–1.59	.3			
LDL-cholesterol	1.12	0.89–1.42	.3			
Mean arterial pressure	1.01	0.99–1.03	.5			
M1	1.28	1.01–1.61	.04	1.3	1.02–1.66	.04
Proteinuria	1.01	0.91–1.12	.8			
Serum creatinine	1.06	0.96–1.17	.3			
Male	1.03	0.57–1.89	.9			
S1	1.96	0.58–6.67	.3			
Total cholesterol	1.02	0.87–1.19	.8			
Triglyceride	1.08	0.92–1.26	.3			
T1	1.53	0.54–4.39	.4			
T2	0.67	0.3–1.49	.3			
Serum IgA	1.01	0.99–1.03	.5			
Uric acid	1.15	1–1.33	.05	1.14	0.98–1.33	.08
Urine red blood cell count	1.01	0.98–1.04	.6			
Renin-angiotensinsystem inhibitor	0.97	0.88–1.06	.5			
Steroid	0.77	0.43–1.37	.4			
Immunosuppressant	0.69	0.37–1.28	.2			

The endpoint was referred to a continuous decline in eGFR of ≥30% from the baseline or reaching ESRD. Factors that showed statistical significance (*p* < .1) in univariate-Cox-regression analysis were chosen to establish a multivariate-Cox-regression model. Subsequently, the AIC using FB stepwise selection was employed to identify the best model with a minimum AIC value.

E1: endocapillary hypercellularity; M1: mesangial hypercellularity; T1-2: severity of interstitial fibrosis/tubular atrophy; S1: segmental glomerulosclerosis/adhesion; C1: presence of crescent; HR: hazard ratio; CI: confidence interval; AIC: Akaike information criterion.

**Table 5.** GLMM analysis for repeated measures of urinary albumin/creatinine ratio between IgAN-NS and IgAN-NR.

	*F*-value	*p*
(Intercept)	303.3	<.001
Cohorts	14.9	.001
Follow-up time	34.2	<.001
Cohorts:Follow-up time	2.68	.04

**Table 6.** GLMM analysis for repeated measures of serum albumin between IgAN-NS and IgAN-NR.

	F-value	*p*
(Intercept)	20361.6	<.001
Cohorts	30.5	<.001
Follow-up time	138.8	<.001
Cohorts:Follow-up time	19	<.001

**Table 7.** GLMM analysis for repeated measures of urinary albumin/creatinine ratio between IgAN-NS and IgAN-NR without CR.

	*F*-value	*p*
(Intercept)	332.5	<.001
Cohorts	20.4	<.001
Follow-up time	13.4	<.001
Cohorts:Follow-up time	6.2	.003

**Table 8.** GLMM analysis for repeated measures of serum albumin between IgAN-NS and IgAN-NR without CR.

	*F*-value	*p*
(Intercept)	11502.8	<.001
Cohorts	39.1	<.001
Follow-up time	62.5	<.001
Cohorts:Follow-up time	1.7	.09

The GLMM was utilized to assess the impact of repeated measures on various factors between the two cohorts and across multiple follow-up periods. The statistical differences were assessed using ANOVA, which enabled the identification of both intergroup/intragroup variations. The cohorts included the IgAN-NS (IgAN with NS) and the IgAN-NR (IgAN with nephrotic-range proteinuria). The interaction between cohorts and follow-up periods was denoted as Cohorts * Follow-up duration. Additionally, CR represented complete remission of proteinuria.

## Discussion

Previously, it was reported that only a small percentage, approximately 5–10%, of IgAN patients presented with NS (referred to as IgAN-NS) [5,7]. In our retrospective analysis of 1870 IgAN patients, we identified 129 patients with IgAN-NS (6.9%). Our study revealed that the clinicopathological features of IgAN-NS were more severe compared to IgAN-NR. In addition to the typical manifestations of NS, such as massive proteinuria, hypoalbuminemia, and hyperlipidemia, IgAN-NS patients exhibited higher levels of inflammatory indicators such as CRP and fibrinogen, as well as more pronounced glomerular mesangial proliferation. These findings suggest a more aggressive inflammatory response and potentially stronger immune-inflammatory reactions in the kidneys of IgAN-NS patients, or a greater susceptibility to infection in individuals with NS. Also, lower hemoglobin levels also indicate more severe disease activity and worse prognosis, which was found in a previous study [20].

We also found that IgAN-NS patients had significantly higher levels of 24-h UP compared to IgAN-NR patients, despite both cohorts presenting with massive proteinuria. These results are consistent with previous findings reported by Li et al. [5] Further investigations are necessary to fully understand the implications of these findings. Our cluster analysis revealed that the correlation patterns of 24-h UP levels differed between the two cohorts, suggesting that massive proteinuria may have distinct clinical significance in each group. In IgAN-NS, the 24-h UP level may correlate with lipid levels, which aligns with the well-known association between NS^11^ and increased secretion of sialylated angiopoietin-like protein 4 (Angptl4) [21], leading to elevated lipid levels. In contrast, in IgAN-NR, the 24-h UP level may correlate not only with inflammatory indicators such as fibrinogen and C-reactive protein but also with serum IgA, urinary RBCs, and WBCs. This suggests that the proteinuria observed in IgAN-NR may be directly associated with the inflammatory damage to the glomerular structure caused by the underlying disease activity itself [22].

Previous research has proposed that the accumulation of IgA-dominant immune complexes in the mesangial and capillary walls can lead to inflammatory damage, subsequently resulting in proteinuria [23]. Based on this information, we speculate that the clinical significance of massive proteinuria may differ between the two cohorts. In IgAN-NR, massive proteinuria may occur at an earlier disease stage where it is primarily related to the aggressive immune-inflammatory response triggered by IgA-dominant immune complexes. However, in IgAN-NS, massive proteinuria may occur at a later disease stage when glomerular mesangial proliferation is more pronounced, and higher levels of 24-h UP contribute to increased lipid levels. At this later stage, proteinuria may not only be influenced by disease activity but also interact with hypoalbuminemia, hyperlipidemia, and other factors, potentially leading to more severe clinical manifestations [10]. These correlations observed in our analysis warrant further confirmation through future studies.

When utilizing a composite endpoint of eGFR decline of ≥30% from baseline or reaching ESRD, our KM analysis revealed no obvious difference in prognosis between the two cohorts after matching gender, age, follow-up time, and eGFR at baseline. Additionally, multivariate Cox regression analysis showed that urine protein and serum albumin were not independent factors associated with the endpoint. These findings are in agreement with a prior study by Li and colleagues [5], which demonstrated that hypoalbuminemia had no direct impact on the prognosis of patients with massive proteinuria. However, since the previous study did not provide follow-up data on serum albumin and proteinuria, further research are needed to draw definitive conclusions. Previous studies have proposed that remission of proteinuria can significantly improve the prognostic outcomes of IgAN patients. Kim and coworkers [7] further observed that IgAN-NS patients with CR had significantly better prognosis compared to those without CR. Therefore, we speculate that the changes in proteinuria during the follow-up may hold great significance for understanding the prognostic features. The GLMM and line charts demonstrated a notable decrease in urinary albumin/creatinine ratio and an increase in serum albumin levels in both cohorts during the follow-up. Additionally, there were no significant between-group variations at the 1-year follow-up, suggesting that the outcomes of proteinuria did not differ significantly between the two cohorts. Statistical results showed that 44.1% of patients in IgAN-NR and 48.6% of patients in IgAN-NS achieved CR, showing no significant difference. However, the inclusion of these patients may not directly reflect the effect of massive proteinuria on prognosis [7]. Therefore, we selected patients without CR from both cohorts to compare their clinicopathological manifestations and prognosis.

Although the cohorts were not matched, there were no obvious disparities in age, gender, follow-up time, and eGFR between the two groups. Notably, IgAN-NS still exhibited more severe inflammation than IgAN-NR and had significantly lower serum IgA levels, further indicating differences in disease activity. KM analysis revealed that in patients without CR, the prognostic outcomes of IgAN-NS were obviously worse than those of IgAN-NR. The GLMM and line charts showed a clear decrease in urinary albumin/creatinine ratio in both cohorts during the follow-up, with no significant between-group variation at the 1-year follow-up, suggesting that the outcomes of proteinuria did not differ significantly between the two cohorts. However, the GLMM and line chart demonstrated a noticeable increase in serum albumin levels in both cohorts, with significantly higher levels in IgAN-NR compared to IgAN-NS during the follow-up. The multivariate Cox regression analysis also revealed that increased serum albumin levels were an independent protective factor. These results suggest that increased serum albumin levels may improve the prognosis of IgAN patients presenting with massive proteinuria but failing to achieve CR. Despite a higher percentage of these patients receiving corticosteroid therapy, the prognosis was not significantly improved, warranting further investigation.

## Conclusion

In conclusion, this study provided preliminary insights into the clinical relevance of massive proteinuria in IgAN-NS and IgAN-NR. In patients without CR, the prognosis of IgAN-NS was markedly worse compared to IgAN-NR, with persistently lower serum albumin levels during the follow-up. Increased serum albumin was identified as a significant protective factor in these patients, complementing the findings of previous studies. However, It is crucial to acknowledge that this study was conducted as a retrospective analysis in a single center, with a limited sample size, potential regional constraints and the small proportion of patients who received EM examination. Additionally, acquiring detailed therapy regimens and long-term follow-up data on proteinuria was challenging. The GLMMs were bulit to reduce the impact of confounding factors such as long-term follow-up and individual differences but it was hard to eliminate the effects of all confounding factors. Therefore, the aforementioned conclusions require further validation through large-sample-size and multi-center studies.

## Supplementary Material

Supplemental Material

## Data Availability

Due to ethical considerations, the data used in this study are not publicly available. For any further inquiries regarding the data, interested individuals can contact the corresponding author.
