# Supplementary material for: Clinical significance of massive proteinuria in primary IgA nephropathy with and without nephrotic syndrome: a single center cohort study
Source: Ren Fail. 2023 Oct 18;45(2):2267138. doi: 10.1080/0886022X.2023.2267138 (PMC10586089; doi:10.1080/0886022X.2023.2267138)
Supplement: Supplemental Material [file IRNF_A_2267138_SM1199.pdf]

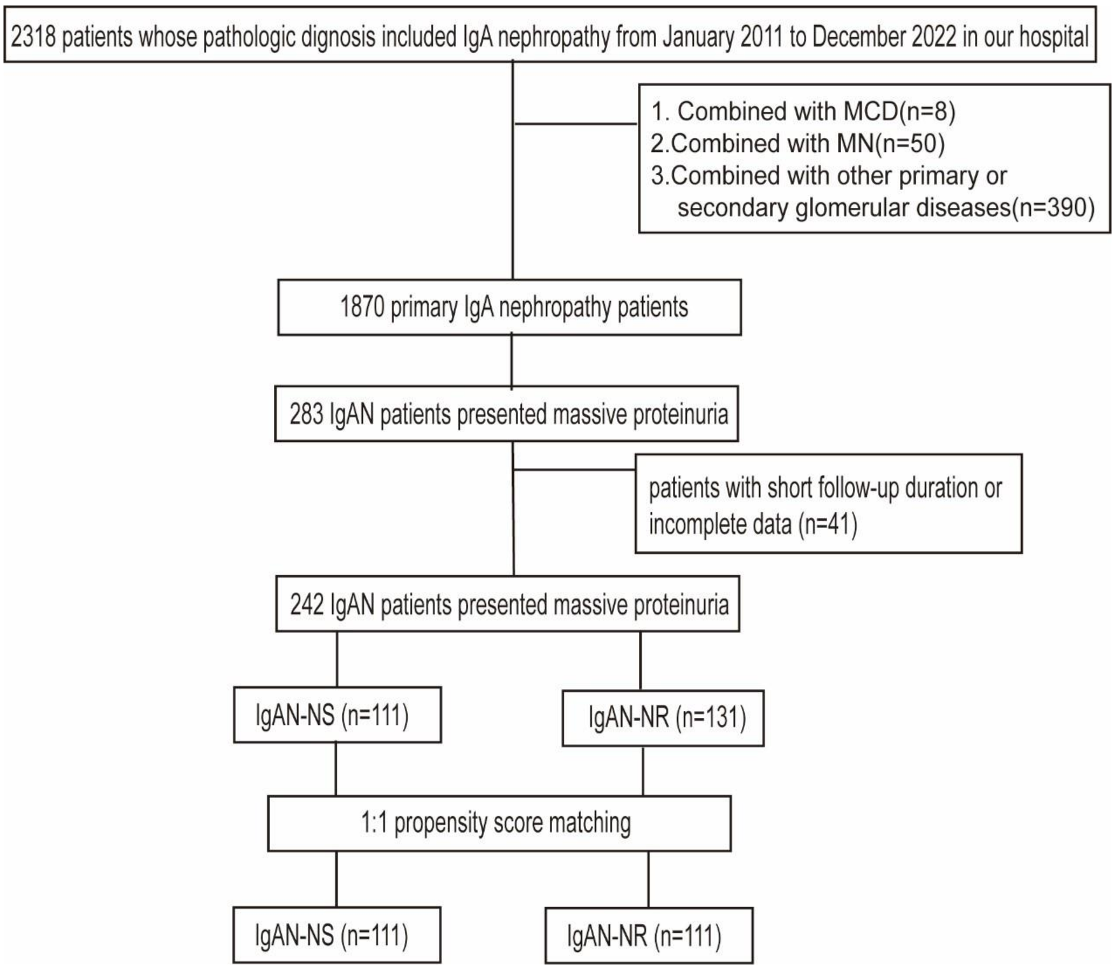


Supplemental Figure 1 The flow chart

IgAN-NS, the IgAN with NS; IgAN-NR, the IgAN with nephrotic-range proteinuria; MCD, minimal change disease; MN, Membranous nephropathy.

Table S1 The percentage of missing data of 222 cases in this study

| Factors | The percentage of missing data |
|---|---|
| Age,y | 0% |
| Male | 0% |
| Follow-up duration,m | 0% |
| eGFR | 0% |
| Body mass index | 2.0% |
| Systolic blood pressure, mmHg | 6.0% |
| Diastolic blood pressure, mmHg | 6.0% |
| Mean arterial pressure, mmHg | 6.0% |
| Hemoglobin, g/L | 2.7% |
| Serum creatinine, mg/dL | 0% |
| Serum albumin, g/L | 0% |
| Total cholesterol, mmol/L | 1.4% |
| Triglyceride, mmol/L | 1.4% |
| HDL-cholesterol, mmol/L | 1.4% |
| LDL-cholesterol, mmol/L | 1.4% |
| Uric acid,mmol/L | 8.1% |
| Fibrinogen, g/L | 2.3% |
| C-reactive protein, g/L | 9.9% |
| Serum IgA, g/L | 1.4% |
| Proteinuria, g/24h | 0% |
| Urine red blood cell count, RBC/μL | 0% |
| Urine white blood cell count, WBC/μL | 0% |
| **Therapy** | |
| Renin-angiotensinsystem inhibito | 0% |
| Steroid | 0% |
| Immunosuppressant | 0% |
